# Implantable Biomaterials for Peripheral Nerve Regeneration–Technology Trends and Translational Tribulations

**DOI:** 10.3389/fbioe.2022.863969

**Published:** 2022-04-27

**Authors:** Angela Sanchez Rezza, Yalcin Kulahci, Vijay S. Gorantla, Fatih Zor, Norman M. Drzeniek

**Affiliations:** ^1^ Charité— Universitätsmedizin Berlin, Corporate Member of Freie Universität Berlin and Humboldt–Universität zu Berlin, Institute of Medical Immunology, Berlin, Germany; ^2^ Wake Forest School of Medicine, Department of Surgery, Wake Forest Institute for Regenerative Medicine, Winston-Salem, NC, United States; ^3^ Berlin Institute of Health at Charité—Universitätsmedizin Berlin, BIH Center for Regenerative Therapies (BCRT), Berlin, Germany; ^4^ Charité — Universitätsmedizin Berlin, corporate member of Freie Universität Berlin and Humboldt- Universität zu Berlin, Berlin-Brandenburg School for Regenerative Therapies (BSRT), Berlin, Germany

**Keywords:** biomaterial, peripheral nerve regeneration, nerve guidance conduit, biofabrication, bioactive material, material structure, growth factors, peripheral nerve injuries (PNI)

## Abstract

The use of autografted nerve in surgical repair of peripheral nerve injuries (PNI) is severely limited due to donor site morbidity and restricted tissue availability. As an alternative, synthetic nerve guidance channels (NGCs) are available on the market for surgical nerve repair, but they fail to promote nerve regeneration across larger critical gap nerve injuries. Therefore, such injuries remain unaddressed, result in poor healing outcomes and are a limiting factor in limb reconstruction and transplantation. On the other hand, a myriad of advanced biomaterial strategies to address critical nerve injuries are proposed in preclinical literature but only few of those have found their way into clinical practice. The design of synthetic nerve grafts should follow rational criteria and make use of a combination of bioinstructive cues to actively promote nerve regeneration. To identify the most promising NGC designs for translation into applicable products, thorough mode of action studies, standardized readouts and validation in large animals are needed. We identify design criteria for NGC fabrication according to the current state of research, give a broad overview of bioactive and functionalized biomaterials and highlight emerging composite implant strategies using therapeutic cells, soluble factors, structural features and intrinsically conductive substrates. Finally, we discuss translational progress in bioartificial conduits for nerve repair from the surgeon’s perspective and give an outlook toward future challenges in the field.

## 1 Introduction

Peripheral nerve injury (PNI) occurs predominantly in the upper limb, including digital nerves and nerves of the brachial plexus and its terminal branches and results in pain, loss of motor functions and sensation of the limb, significantly impacting the patient’s quality of life (Ciaramitaro et al., 2010). PNI occurs most often due to trauma, but also as a consequence of metabolic diseases such as diabetes (Sahin et al., 2012), nerve compression such as carpal tunnel syndrome or even vessel thrombosis. In most cases other tissues are affected by the injury as well, but even if an amputated limb can be replanted micro-surgically, the functional outcome of the limb is often limited by the degree of nerve injury and its capacity to regenerate.

PNI regeneration is often categorized by the Sunderland classification, where grade I is defined as a defect of the myelin layer without any damage to the axons and grade II describes injury to the axons without disruption of supporting connective tissue sheaths such as the epi-, peri-, and endoneurium. Grades III-V describe additional injury to the endoneurium, perineurium, or complete transection of the nerve and are associated with significantly worse healing outcomes (Sunderland, 1951). This observation highlights the outcome-determining role that the (micro-) anatomical guiding sheaths play in nerve regeneration and reconstruction.

Axonal injury triggers a series of events especially at the distal nerve segment called Wallerian degeneration, which is driven by Schwann cells. Activated Schwann cells increase their mitotic rate with upregulation of several genes to orchestrate the degeneration and repair process. Following migration of macrophages, Schwann cells and macrophages work together to clear all myelin lipid and other axonal debris and prepare the nerve for regeneration (Burnett and Zager, 2004).

Proliferating and migrating Schwann cell later develop glial bands of Büngner which encase basal lamina. These bands provide neurotrophic and structural support and guide the regrowing axon back to innervate its former target (Stoll and Müller, 1999; Burnett and Zager, 2004; Namgung, 2014). Although the regeneration process of the nerve seems to be a standard sequence of events, there are many variations in the detail, especially in the regeneration of sensory and motor nerves. There are intrinsic (the embryonic origin) and extrinsic (environmental) differences between these two fiber types causing different regenerative capacities. Although activation of class II and III b-tubulin genes and downregulation of neurofilament genes NF-L, NF-M, and NF-H are common in both type of fibers, their cytokine milieu is different: IL6, IL1b and TNFa and LIF are more prominent in sensory fiber regeneration while NGF-R, trkB, BDNF and NT-4 are more prominent in motor fiber regeneration (Stoll and Müller, 1999). To differenciate in detail between motor and sensory nerve regeneration is beyond the aim of this article, but because most nerves are a combination of these two fibers, a strategy which enables regeneration of both fiber types is needed.

Indeed, although peripheral nerves possess the theoretical ability to regenerate at a rate of about 1 mm/day, successful regrowth is dependent on surgical reconstruction of these anatomical guiding sheaths (Seddon et al., 1943). However, unless neurorrhaphy is performed within a day after injury, the stumps of a transected nerve recoil and a gap is formed, making tension-free coaptation impossible. A graft is then needed to bridge the anatomical gap (Sahin et al., 2014).

A “critical nerve gap” is defined as a nerve gap over which no recovery will occur without nerve grafting or bridging. It is generally accepted that all vertebrate species posess the same velocity of nerve regeneration. However, intrinsic differences of each species result in different nerve gap size being considered critical. In rats, the critical nerve gap is considered ∼1.5 cm, in rabbits ∼3 cm, and in pigs and humans ∼4 cm (Kaplan et al., 2015).

While in terms of healing outcome the autograft approach is still the gold standard to bridge a critical gap defect, it creates another nerve defect at the donor site and in most cases consists of a small-diameter cutaneous nerve unsuitable to repair large-diameter nerves. Therefore, there is a need for bioartificial, off-the-shelf nerve guidance conduits (NGCs) that promote nerve regeneration. In this review we provide an overview of classic and emerging conduit design strategies, highlight how principles derived from the regenerative medicine field are being used to augment bioactivity of the implants and discuss their potential value for clinical practice.

### 1.1 Bioartificial Nerve Grafts Available on the Market

Years of preclinical research in nerve regeneration have resulted in only few products on the market. Perhaps the most popular and widely accepted one is the decellularized allograft from deceased human donors, known as Avance (AxoGen, Alachua, FL). Although the use of AxoGen eliminates donor site morbidity, it has the limitation of usage of biological material that can be characterized only to a certain extent (Rbia et al., 2019). Another product developed by Integra Life Sciences (Plainsboro, NJ) and composed of semi-permeable Type I collagen is marketed under name Neuragen. A practical limitation of tubular collagen products is their tendency to collapse and kink and their potential for scarring (Kornfeld et al., 2021a).

Currently, more biocompatible synthetic polymer product made from polyglycolic acid (PGA) or poly-lactidecaprolactone (PLCL) are gaining popularity in nerve gap repair. Neurotube is produced from woven polyglycolic acid (PGA); by Synovis Micro Companies Alliance (Birmingham, AL) while Neurolac is a PLCL conduit (Hussain et al., 2020). The newest FDA approved product is Nerbridge, which is composed of polyglycolic acid and collagen derived from porcine skin. This product is composed of resorbable and semipermeable tubular membrane matrix filled with porous collagen and provides a non-constricting encasement for injured peripheral nerves (Matsui et al., 2016). Despite many advantages of synthetic materials, their biologcial performance cannot reach allogenic nerve grafts. Therefore, they have only limited application for repair of short gaps (Kaplan et al., 2015).

## 2 Translational Design Criteria for Nerve Guidance Channels

**FIGURE 1:**
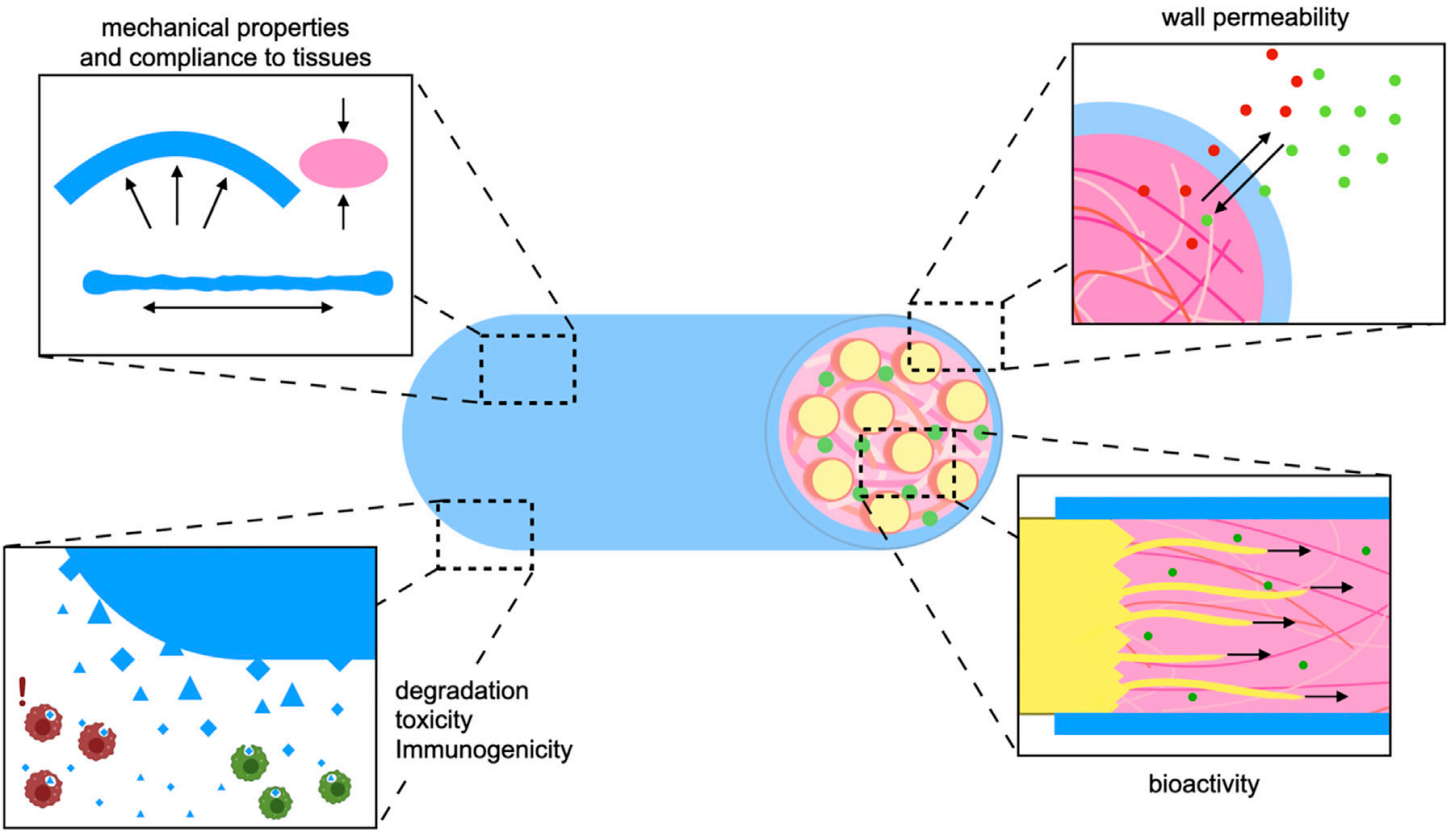
Design criteria for nerve guidance implants: For the development of effective synthetic nerve grafts biomaterial and biofabrication strategies should be applied in the service of established design criteria. The implant needs to comply with living tissue but possess enough strength and stability to withstand forces resulting from joint and muscle movement (top right). In order to avoid a surgical removal, the implant should degrade biologically and its degradation products must not harm the regenerating nerve nor other surrounding tissues (bottom right). The conduit wall should be permeable enough to allow for entry of nutrients and efflux of metabolic waste. At the same time the conduit wall creates a compartment that retains biochemical cues and soluble growth factors at the site of regeneration (top left). In order to bridge larger nerve gap injuries the implant should actively promote axon growth in a setting where regeneration would be otherwise ineffective or take too long. This can be achieved both by intrinsic properties of bioactive polymers, as well as their functionalization through biochemical or physical cues (bottom left).

With experience gained from over three decades of translation-oriented development and *in vivo* evaluation of NGCs, several general requirements and desirable properties have been identified (Figure 1).

- Sufficient availability: The limited availability of natural grafts, such as autografts, allografts or xenografts is a major incentive to develop bioartificial NGCs.
- Size/diameter: In contrast to naturally harvested grafts, bioartificial implants can be tailored to match a specific defect or the diameter of the nerve to be reconstructed.
- Mechanical strength: The tensile strength of a peripheral nerve lies in the megapascal (MPa) range (Borschel et al., 2003). Ideally, the conduit should match the mechanical properties of the native nerve (Figure 1). The NGC wall should be strong enough to allow for suturing and to prevent collapsing, but too much rigidity can cause trauma to surrounding tissues during movement of joints and muscles. For example, empty vein grafts may kink and collapse across larger defects (Tang et al., 1995).
- Biodegradability: If the implanted material is not degradable it needs to be removed surgically in time before it starts to compress the newly sprouted axon bundles. Silicone tubes are an early historic example of non-degradable synthetic conduits. Since then, biodegradable polymers have been investigated for PNI repair (Weber et al., 2000). Because depending on defect size healing might take more or less time, the degradation rate, which is depends on the polymer used and on the micromilieu at the implantation site, should also be considered, as discussed by Barrows (1986). So far there is no consensus on the ideal degradation time of an NGC *in vivo*.
- Toxicity/immunogenicity: When implanting degradable biomaterials *in vivo*, no matter if natural or synthetic, low toxicity of degradation products should be a top priority (Figure 1). Additionally, the immune response to the graft might cause excessive inflammation which hampers successful healing in different tissues (Reinke et al., 2013; Benga et al., 2017).
- Wall permeability: The conduit wall should let nutrients in and waste metabolites out (Figure 1). However, a certain degree of compartmentalization is necessary to prevent scar tissue and inflammatory cells from entering the lumen (Zor et al., 2014).
- Bioactivity: Bioactive substances can actively support neuron survival and axon attachment, reduce scarring or inflammation, and promote axon sprouting or vascularization (Benga et al., 2017; Yapici et al., 2017). As critical-size defects still pose an unsolved problem, current NGC design should aim at incorporating biochemical, mechanical, and structural bio-instructive cues into the implant without sacrificing any of the criteria listed above (Figure 1).

## 3 Substrate Choice

### 3.1 Synthetic Biodegradable Polymers

To date aliphatic polyester NGCs are the only fully synthetic NGCs with FDA approval.

In contrast to naturally harvested materials such as the autograft or NGCs fabricated from naturally occurring proteins, their chemically defined synthesis, well understood *in vivo* performance and superior control over material properties allow for mass production and commercialization, providing a potential alternative in surgical management of PNI. While the exact degradation time depends on polymer formulation, implant size and location and its surface exposure to water, polyglycolic acid (PGA), polylactic acid (PLA), polycaprolactone (PCL) and copolymers such as polylactide-co-glycolide (PLGA) or polylactide-co-caprolactone (PLCL) are all hydrolytically degraded within weeks to months and absorbed in a controlled process that is accompanied by only a moderate inflammatory reaction (Pavan et al., 1979; Barrows, 1986; Ginde and Gupta, 1987).

Because the presence of methyl groups results in stronger hydrophobicity, hydrolytic degradation of PLA takes longer than for PGA. Depending on the expected regeneration rate this can be an advantage as rapid degradation could lead to premature NGC breakdown and loss of mechanical strength (Ginde and Gupta, 1987; Matsumine et al., 2014). On the other hand, continuous degradation is needed to gradually relieve pressure on regenerated axons. A potential solution to this dilemma, the copolymer PLGA has been found to degrade faster than PGA or PLA but retained strength longer (Craig et al., 1975). The degradation and mechanical strength of PLGA can be further adjusted by changing the PLA/PGA ratio (Makadia and Siegel, 2011).

Biodegradable polyesters were promising early candidates for NGC design because in contrast to a silicon tube they eliminated the need for surgical removal. Weber et al. (2000) found that a PGA-based NGC commercialized as NeurotubeTM led to better functional recovery than autograft after digital nerve injury which is among the most common types of PNI (Ciaramitaro et al., 2010). Key principles of structural design are found in this early product: the corrugated wall prevents collapsing of the tube *in vivo*, while porosity allows for an oxygen-rich environment (Dellon and Mackinnon, 1988; Weber et al., 2000; Rosson et al., 2009).

Despite early commercialization and some positive results in the repair of short nerve gaps, the aliphatic polyesters did not lead to a breakthrough in the field of surgical nerve repair due to their lack of bioactive cues that could support nerve growth across larger gaps. Bioactive proteins such as growth factors or adhesion ligands are difficult to incorporate as the high process temperatures of the polymers lead to protein degradation (Benga et al., 2017). In the following sections we will discuss strategies that aim at combining advantages of synthetic polymers and bioactive materials.

### 3.2 Bioactive Natural Polymers

The success of the autograft is likely attributable to the presence of Schwann cells and growth factors as well as attachment sites and guiding sheaths for the sprouting axon. These properties are most easily recapitulated using naturally occurring biopolymers like polysaccharides (HA, chitosan) or proteins (collagen, gelatin, silk). Some of these natural polymers like collagen and laminin are physiologically present within the nerve and are natural candidates for re-engineering the damaged microanatomy (Wallquist et al., 2002). Other naturally occurring polymers such as silk, gelatin or chitosan can interact with human tissue and provide bioactive cues for healing although they are not physiologically present within the human body.

Because natural polymers need to be harvested form animal sources, their purity and their physicochemical and immunological properties are more difficult to control, making translation potentially challenging. An alternative to harvested materials are recombinant sources, which offer potentially well-defined biopolymers with minimized impurity, but are significantly more costly and require advanced production methods. For example, production of recombinant collagen from bacterial, yeast or mammalian cells allows to recreate a variety of its native structures or even introduce new derivatives. Despite these advancements and great demand for collagen in both the biomedical and the food industry, both industries still rely on animal-derived sources, because there is no consensus on the preferred structure or production method and production of recombinant protein remains more expensive than the well-established harvesting from animal herds, as discussed in detail by Fertala (2020) and colleagues.

#### 3.2.1 Decellularized Extracellular Matrix Allograft

As an alternative to the autograft, allografts from cadaveric donors could avoid donor site morbidity but would be rejected by the host immune system due to human leukocyte antigen (HLA) mismatch. To avoid graft rejection, the tissue needs to be decellularized, removing the HLA epitopes presented on the cell surface but processed nerve allografts maintain the extracellular matrix and its structure, which is advantageous as discussed in Section 4.3 (Whitlock et al., 2009). Cell removal can be achieved by chemical processing with a detergent or physical methods such as irradiation, lyophilization or thermal treatment. Moore and colleagues showed that the processing method has an influence on graft performance, with chemical decellularization leading to better graft function than physical processing (Moore et al., 2011).

Decellularized allograft resulted in regeneration superior to bioartificial collagen-based conduits, but still inferior to autograft in rat models (Whitlock et al., 2009; Giusti et al., 2012). More recently however, a direct comparison in digital nerve repair in human patients with a commercially available collagen NGC (NeuraGen by Integra) versus an allograft (Avance by AxoGen) showed similar results for both products (Rbia et al., 2019). However, both products failed to achieve an outcome that was evaluated as excellent in most patients. This and further limitations related to availability and storage of allografts as well as the high cost, create a strong incentive to engineer synthetic NGCs that can be mass produced and are available off the shelf at different sizes, sufficient quantity and accessible cost.

#### 3.2.2 Collagen and Gelatin

The most abundant extracellular matrix (ECM) protein Collagen type I is widely implemented as a biomaterial for tissue repair. Collagen consists of a triple helix of three polypeptide chains. Fibrils are formed by cross-links between telopeptides that contribute to the relatively high moduli of telo-collagen, as discussed in depth elsewhere (Gelse et al., 2003).

Collagen provides attachment sites for neurons, that have been shown to contribute to neurite outgrowth (Ivins et al., 2000). Additionally, collagen can act as a scaffold for growth factors and cytokines and thus play an important role in mimicking a biologically relevant healing environment.

A collagen nerve guidance conduit was developed for the regeneration of a 4 mm nerve gap and demonstrated comparable nerve growth to the autograft method in rats and monkeys (Archibald et al., 1991). This product was FDA-approved and commercialized under the name NeuraGen and allowed for regeneration of nerve gaps of 6–18 mm in human patients (Lohmeyer et al., 2007).

As gelatin is denatured collagen that lacks the triple helix structure, it seems that it could be used for NGC construction as well. However, its weak mechanical properties and its tendency to move quickly away from the implant site make additional modification and cross-linking necessary to increase mechanical stability of the NGC (Ko et al., 2017). A clear advantage of both collagen and gelatin is their biodegradability by enzymes present in the healing milieu (van Amerongen et al., 2006; Kuwahara et al., 2011).

#### 3.2.3 Laminin

Laminin, a glycoprotein of the basal lamina secreted by Schwann cells, has been described as a particularly favorable substrate for adhesion, migration, and regeneration of axons (Mammadov et al., 2016). Laminin is naturally present within the lesioned nerve and exerts its function through interaction with integrin receptors that are upregulated on the neuron cell membrane upon PNI such as integrins α6β1 and α7β1 (Wallquist et al., 2004; Gardiner et al., 2005). The 18 laminin isoforms have varying affinities for different integrins, which have been reviewed in detail elsewhere (Nieuwenhuis et al., 2018). Rather than as a base material, laminin is used in the tissue engineering field to functionalize other synthetic or natural polymer networks (Barros et al., 2019; Drzeniek et al., 2021). Drzeniek et al. (2021) have demonstrated covalent linking of laminin into a methacrylated collagen hydrogel, which improved the material’s bioactive properties. Chang et al. (2020) have recently used laminin to functionalize a synthetic PCL NGC, demonstrating its use as a pro-regenerative additive in PNI.

#### 3.2.4 Hyaluronic Acid

HA is a highly hydrophilic glycosaminoglycan of the ECM, that contributes to the cushioning function of cartilage due to its high-water content. Already applied clinically in several indications reviewed by Abatangelo et al. (2020) HA is suitable for implantation, injection and tissue engineering purposes due to its viscoelastic properties, biocompatibility, biodegradability and bioactivity. In the field of peripheral nerve regeneration, hyaluronic acid was used as a conduit filler to facilitate axon migration and myelination in the regeneration of a 10 mm rat sciatic nerve gap (Wang et al., 1998). HA can also be implemented to overcome extraneural scarring, as it has been shown to reduce adhesion of the nerve to the neural bed (Ikeda et al., 2003; Zor et al., 2014).

#### 3.2.5 Chitosan

Chitosan is a linear polysaccharide derived from chitin, the major component of the exoskeleton of crustaceans and insects, through chemical or enzymatic processes. Its reactive groups make chitosan accessible to a myriad of chemical modifications and biofabrication techniques, resulting in fibers, beads, films, gels, scaffolds or nanoparticles (reviewed by El Knidri et al. (El Knidri et al., 2018)). Additionally, chitosan possesses an antimicrobial activity probably due to its positive charge and interactions with the negatively charged cell membrane and thus could be a useful component of implantable biomedical devices (D'Almeida et al., 2017). In peripheral nerve regeneration the positive charge of chitosan interacts with the negatively charged axons and significantly improved functional outcome in human patients in a randomized controlled trial of primary surgical nerve repair (Neubrech et al., 2018). In preclinical models chitosan has also been shown to improve regeneration of critical sized nerve injuries (30 mm dog sciatic nerve) and to reduce neuroma formation and fibrosis (Wang et al., 2005; Marcol et al., 2011). Limiting to the use of chitosan are its weak mechanical properties and the low mechanical strength of chitosan is even reduced in physiological environments (Madihally and Matthew, 1999; El Knidri et al., 2018). This challenge can be overcome by additional chemical modification, cross-linking or hybrid use of chitosan with other materials. Thus, most recent NGC designs implement combinations of chitosan and collagen or gelatin (Singh et al., 2019; Itai et al., 2020).

#### 3.2.6 Silk

Silk is a protein fiber produced by insects, among others silkworms, spiders and bees, to construct webs and cocoons. Its major component is silk fibroin, a semicrystalline protein, which has been studied for peripheral nerve applications mostly in the form of a silk fibroin protein solution from the silkworm *Bombyx mori* or the spider *Nephila clavipes* (Radtke et al., 2011; Radtke, 2016). Silk has been FDA-approved for decades and is used as a surgical suture material. In contrast to the other natural biomaterials mentioned above, silk convinces due to its extremely high tensile strength paired with natural proteolytic degradability (Wongpinyochit et al., 2018). Importantly, silk is biocompatible with nerve and has been shown to support attachment and survival of neurons and Schwann cells not only *in vitro* (Yang et al., 2007) but also in a large animal model (Radtke et al., 2011).

The protein structure of silk can be modified through genetic engineering and its many functional groups offer a myriad of opportunities for functionalization with additional bioactive domains or growth factors (Kong et al., 2020). In a recent study, a silk fibroin-HA-composite matrix was proposed and implanted subcutaneously into immunocompetent mice to assess immunogenicity. The addition of silk fibroin to HA resulted in a faster regrowth of blood vessels and synthesis of new ECM compared to HA alone, without triggering any excessive inflammation. This demonstrates that silk could be used in multimodal NGC designs to improve the bioactivity and mechanical strength of other materials (Gisbert Roca et al., 2020). The modifiable properties of silk can be implemented for functionalization with neurotrophic factors. A controlled-release-NGC implant successfully restored motor function and reduced distal muscle atrophy in a 10 mm rat sciatic nerve gap study, as the controlled release of GDNF from the proximal conduit led to retrograde neuroprotection not noticed in the plain silk conduit group (Carvalho et al., 2021).

Beyond preclinical rodent models, Kornfeld et al. (2021b) have recently demonstrated that spider silk nerve implants can support axonal regeneration in a 6 cm nerve defect in adult sheep with comparable efficacy to autologous nerve grafts. This result represents one of the most translationally mature implementations of a biomaterial graft in a large nerve gap and paves the way for translation of silk-based conduits into clinical practice.

### 3.3 Conductive Materials

As an electrified tissue, the peripheral nerve offers the opportunity to be stimulated through electrical cues, in addition to classical soluble or material-mediated bioinstructive cues used in other areas of tissue engineering and regenerative medicine. Conceptually, this can involve the use of intrinsically conductive organic polymers as a substrate for electric communication between cells or the inclusion of soft neural interfaces and electrode arrays with read or write functionalities (Kim et al., 2015; Chen et al., 2019; Paggi et al., 2021). In addition to implantable biomaterial strategies, electrical stimulation of the healing nerve is a clinically validated procedure which could be applied in synergy with an NGC strategy and has been reviewed elsewhere (Gordon, 2016).

#### 3.3.1 Intrinsically Conductive Polymers

Organic polymers such as polyaniline, PEDOT and polypyrrole owe their electrical properties to their conjugated bonds as discussed by Le and colleagues (Le et al., 2017). When included in an NGC without an external electricity source, these materials do not actively actuate the healing nerve, instead they can respond to and conduct electrical activity of interfacing neurons. Recently, Vijayavenkataraman et al. (2019)a have demonstrated that neural crest stem cells on conductive a polypyrrole-PCL substrate differentiated into peripheral neurons without any external electrical stimulation. The advantage of these organic polymers is their biocompatibility, the disadvantage being their lack of biodegradability and biochemical motifs that can be recognized by cells. To overcome the latter issue, Khor and colleagues attempted almost three decades ago to impregnate entire animal tissues with polypyrrole, but found that the coated tissues did not conduct electricity, likely due to the discontinuity of the polypyrrole in the surface layer of the tissue (Khor et al., 1995). To integrate the conductive polymers homogenously into a biologically relevant ECM, more recent studies blend them with collagen or other biopolymers prior to gelation (Vijayavenkataraman et al., 2019b; Zarei et al., 2021). With increasing proportions of the conductive polymer, conductivity improves, while biological properties are reduced. To add biological cues without disrupting conductivity, conductive polymers can also be functionalized with cell-adhesive peptides such as the laminin-derived YIGSR motif (Green et al., 2009). Alternatively, biodegradability can be introduced, by co-polymerizing the conductive polymer with a biodegradable polymer, instead of blending (Durgam et al., 2010; Vijayavenkataraman et al., 2019b). Recently conductive polymers have been gaining attention in the nerve regeneration community and the implementation of different combinations of conductive materials in NGCs has been shown to enhance axonal growth *in vivo* (Sun et al., 2019)*.* Furthermore, Zhao and colleagues have shown that not only neurons, but also Schwann cells (SCs) respond to the electrical stimulation (Zhao et al., 2020).

#### 3.3.2 Neural Interfaces

Peripheral nerve interfaces’ primary function is to interrogate or actuate the peripheral nervous system with electrode arrays for applications such as neuropathic pain management, nerve recording for limb prosthetics or replacement of peripheral nerve function for bladder control, as reviewed by Paggi and colleagues (Paggi et al., 2021). However, depending on their design and geometry, the interfaces may be used in synergy with a regenerative implant to actively stimulate regenerating axons or monitor healing success both as a research question and as a medical theranostic device. Such strategies involve either a sieve electrode which allows for the passage of growing axons (Lago et al., 2005; MacEwan et al., 2016) or a multichannel electrode conduit (Musick et al., 2015). Although implantable nerve interfaces often face similar problems as NGCs, including mechanical compatibility, biocompatibility and immunogenicity, the material choice for neural interfaces is not typically motivated by questions of bioactivity. Instead, a metal electrode such as gold or platinum is supported by a bioinert substrate such as silicone or polyimide (MacEwan et al., 2016; Paggi et al., 2021). Approaches that combine a neural interface with neuroregenerative functionality would have to draw from both research fields, pairing a spatiotemporally precise interface with a bioactive and biodegradable polymer.

## 4 Biological Functionalization Beyond the Hollow Tube

A possible middle ground between the translatability of synthetic polymers and the biological advantages of natural polymers is the controlled bio-functionalization of well defined NGCs. When a PNI occurs, neurotrophins, chemoattractants and pro-angiogenic factors are naturally secreted in increased amounts, each targeting different mechanistic aspects of nerve regeneration. Thus, studies on advanced NGCs often aim to combine the desirable physicochemical properties of a polymer with defined bioactive agents such as soluble growth factors or cells. Additionally, recent studies demonstrate the use of structural features and electrically conductive materials to promote and guide nerve regeneration.

### 4.1 Functionalization With Soluble Factors

Soluble factors that affect nerve regeneration have been reviewed in detail elsewhere (Benga et al., 2017). Here we give an overview of functional classes of relevant factors and how they are implemented in an NGC approach.

#### 4.1.1 Neurotrophins

As the name suggests, neurotrophins such as NGF, BDNF or NT-3, exert primarily trophic effects on neurons, although some axon guiding effects have also been discussed (Guthrie, 2007; Lykissas et al., 2007).

The most studied member of the neurotrophin family, Nerve Growth Factor (NGF), acts mostly on sensory and sympathetic neurons by promoting neurite sprouting and elongation. Xia and Lv, (2018) loaded an electrospun nanofibrous scaffold with VEGF and NGF, allowing NGF to be released continuously for more than a month. The NGF condition induced stronger proliferation of neural crest stem cells *in vitro* and better functional recovery after sciatic nerve gap injury *in vivo*. NGF was also tested as an axon guidance molecule, showing a chemoattractant function on growth cones *in vitro* (Dontchev and Letourneau, 2002). However, this effect could not be reliably confirmed in a milieu required for ganglionic cell growth, resulting in no directionality and disorganized growth (Fornaro et al., 2020). Brain Derived Neurotrophic Factor (BDNF) is known for its involvement in hippocampal neurogenesis and its protective role in neuronal survival after a PNI, as higher expression of BDNF in Schwann cells and dorsal root ganglia (DRG) were detected after PNI (Kobayashi et al., 2008). Lopes et al. (2017) used tetanus toxin-conjugated nerve targeting nanoparticles to overexpress BDNF DNA in a nerve crush injury model. Their approach led not only to a significantly higher count of myelinated axons but also protected the denervated muscle. This shows that neurotrophins can be valuable therapeutic targets for PNI both on a protein and a gene therapy level.

Neurotrophin-3 (NT-3) can enhance Schwann cell migration and ensure their survival. Donsante et al. (2020) showed that a continuous release of NT-3 from collagen-based electrospun fibers over a 2 week-period led to increased axon counts in the distal nerve. In a co-culture of DRG-derived neurons and then in dorsal root ganglia (DRG) explants NT-3 ensured ordinate and oriented axonal elongation (Fornaro et al., 2020). These recent studies indicate NT-3 as a potential axon guiding component for next generation anisotropic NGCs, although further studies are needed to confirm its axon guiding function *in vivo* and determine the optimal time and mode of delivery.

Glial cell line-Derived Neurotrophic Factor (GDNF), a neurotrophic factor produced by Schwann cells (SCs) has been shown to promote the survival of sensory neurons and axon outgrowth from DRG explants *in vitro* (Leclere et al., 1997) but when released at high concentrations from an NGC it impeded nerve regeneration *in vivo* (Kong et al., 2021). Kong and colleagues discuss this unexpected observation in the context of the “candy store effect,” a term used to describe the entrapment of growing axons in a microenvironment oversaturated with growth factor (Eggers et al., 2013; Kong et al., 2021). It has been suggested that to harness the pro-regenerative function of GDNF a concentration gradient or continuous delivery at low levels via gene therapy is needed (Shi et al., 2010; Eggers et al., 2013).

This last example reminds us, that observations from *in vitro* studies on neurons or explanted dorsal root ganglions (DRG) treated with recombinant growth factors cannot always be directly translated into conclusions about the factor’s beneficial role in a therapeutic application. The optimal dose, timing, release kinetic and localization may be entirely different for each therapeutic protein and the current challenge lies in understanding and modulating the application-specific pharmacokinetics and pharmacodynamics. While there is a strong consensus on the pro-regenerative potential of soluble factors such as neurotrophic growth factors or the matrix remodeling enzyme chondroitinase in PNI, these potent factors are not a one one-size-fits-all solution and their biological function is dependent on the timing, microanatomical localization and often gradient.

#### 4.1.2 Chemoattractant Gradients

An anisotropic aspect could be added to NGC implants by exposing growth cones to chemical gradients of chemoattractants that determine growth directionality rather than growth rate only. Netrin-1 (Ntn1) and its receptor “Deleted in colorectal carcinoma” have been shown to attract the tip of a sprouting axon *in vitro* and promote peripheral nerve regeneration, as well as Schwann cell proliferation and migration (Lv et al., 2015; Wang et al., 2019). Other classes of molecules, such as slits act as axon repellent cues, preventing disorganized growth (Long et al., 2004). Lykissas et al. (2007) discussed that while axon attracting effects have also been reported as an additional function for some members of the neurotrophin family, other classes of soluble molecules that predominantly guide axon growth cones constitute a novel and largely unexplored opportunity in the NGC field. Ntn1 was first used for NGC design in a recent study by Huang and colleagues. A graphene mesh-supported double-network hydrogel scaffold was engineered in which Ntn1 promoted Schwann cell migration successfully and guided their alignment, outperforming even the autologous graft (Huang et al., 2021). Thus, chemoattractant agents should be investigated in further studies, as many *in vitro* findings on axon guidance are yet to be validated in an *in vivo* PNI model.

#### 4.1.3 Adjuvant Soluble Factors

Because every growing tissue is dependent on nutrient supply, sufficient vascularization has an important supportive role in the process of nerve regeneration. As Yapici et al. (2017) have shown, vascularized conduits significantly outperformed non-vascularized conduits in a sciatic nerve gap model. Therefore, Vascular Endothelial Growth Factor (VEGF) was used in combination with mesenchymal stem cells (MSCs) to functionalize autogenous vein grafts. The VEGF group resulted in a higher degree of regeneration of a 10 mm nerve gap, compared to MCSs alone (Eren et al., 2016). Beside its pro-angiogenic activity, VEGF can stimulate axonal outgrowth and promote Schwann cell proliferation and migration, suggesting an entire palette of additional mechanisms through which VEGF could enhance nerve regeneration (Sondell et al., 1999; Muratori et al., 2018).

If the PNI is older and repair occurs with a significant delay, a glial scar consisting of chondroitin sulfate proteoglycans (CSPG) and glycosaminoglycans can interfere with axon regeneration even after successful readaptation of transected nerve ends. Chondroitinase ABC, an enzyme derived from the bacterium Proteus vulgaris, catalyzes the degradation of the polysaccharides. Enzymatic removal of the CSPG scar by chondroitinase treatment is an established strategy in the field of spinal cord repair, but also in PNI (Kostereva et al., 2016; Donsante et al., 2020). Chondroitinase may be an especially valuable factor in a setting of delayed repair because its mode of action could complement the pro-regenerative or trophic approaches discussed above. A recent study has identified an additional mechanism through which the enzyme can disinhibit a BDNF receptor, leading to increased neuroplasticity (Lesnikova et al., 2021).

### 4.2 Functionalization With Transplanted Cells

Aside from the sprouting axons of the injured nerve, different cell types play a role in PNI and can support regeneration. In the NGC approach, the goal is not to grow new neurons in the nerve gap, but instead to regenerate the neurites of lower motor neurons and sensory DRG neurons, the cell bodies of which are located proximal to the injury. Transplanted cells can provide a supportive structure for axons, secrete cytokines, or be engineered to produce and release specific paracrine factors. The challenge for most cell types lies in securing a reliable cell source that ensures controllable product quality, minimal batch to batch differences and off-the-shelf availability. The therapeutic cells’ functionality depends strongly on their microenvironment and can be harnessed by considering cell-material interactions in the NGC design strategy (Keshavarz et al., 2020; Panzer et al., 2020; Drzeniek et al., 2021).

#### 4.2.1 Schwann Cells

SCs are the principal glial cells of the peripheral nervous system (PNS) and ensure the formation of myelin sheaths around the axons and the accelerated conduction of nerve impulses as reviewed elsewhere (Berrocal et al., 2013; Fallon and Tadi, 2021). A recent study shows that SCs exist in functionally diverse differentiation states, as they can dedifferentiate into a precursor-like, proliferating state, which can downregulate myelin genes and remove pre-existing myelin debris that would inhibit axonal regrowth (Arthur-Farraj et al., 2012; Stratton et al., 2018). Beside their ability to myelinate regenerated axons, SCs enhance axonal sprouting and form bands of Büngner, important guiding structures for the growing axon. To mimic the bands, SCs could be compressed into bundles and surrounded by a hydrogel microcolumn. Such tissue engineered Büngner bands accelerated axonal growth *in vitro* up to 8 fold and resulted in significantly longer neurite length than an NGC with unaligned SCs (Panzer et al., 2020). An early study of SCs for PNI treatment reported that *in vitro* cultured SCs would align along the axis of the NGC in structures reminiscent of Büngner’s bands. The same study also found that only syngeneic SCs promoted the growth of myelinated axons, while heterologous SCs induced a host immune response that impaired regeneration (Guénard et al., 1992). The importance of autologous SC sources poses a considerable challenge in terms of NGC availability, standardized product quality control and mass fabrication.

#### 4.2.2 Induced Pluripotent Stem Cells

iPSCs are a personalized self-renewing source of stem cells which enables autologous treatment. iPSCs can be reprogrammed from patient-derived somatic cells and then differentiated into a broad spectrum of cell types, enabling researchers to generate large quantities of cells that are difficult to harvest, such as SCs (Takahashi et al., 2007). On the downside, iPSCs’ pluripotent state has demonstrated the risk of teratoma formation if some undifferentiated iPSCs remain in the transplanted iPSC-derived cell population. Different strategies, including cell sorting or the introduction of suicide genes into iPSCs prior to differentiation are being investigated to provide the safety, necessary for human application of iPSC-derived cells (Bedel et al., 2017). For preclinical evaluation in PNI, iPSCs were differentiated into neural crest stem cells (NCSCs) or SCs without teratoma formation. Huang et al. (2017) used these two cell types to create a tissue-engineered NGC that successfully regenerated a 10 mm gap in a rat model. NCSCs, showed stronger paracrine signaling than the further differentiated SCs, pointing out the importance of selecting the iPSC differentiation state.

Because generating patient-derived iPSCs and differentiating them into a desired cell type takes time, their usefulness for PNI management is limited. Strategies for off-the-shelf clinical use and for complete depletion of undifferentiated iPSCs needs to be developed to fully unleash their potential.

#### 4.2.3 Mesenchymal Stromal/Stem Cells

MSCs are adult multipotent stromal cells isolated from bone marrow, adipose or perinatal tissues. MSCs have attracted attention due to their well-documented clinical safety, rich pro-regenerative secretome, and immunomodulatory properties (Drzeniek et al., 2021). Additionally they can differentiate into SC-like cells when exposed to a combination of soluble factors and produce even more growth factors in this pre-differentiated state (Drzeniek et al., 2021; Liu et al., 2022). Despite many functional similarity and a similar mode of action, MSCs derived from different sources differ in characteristics such as expansion speed and hemocompatibility, as reviewed by Moll and colleagues (Moll et al., 2019).

Intravenously infused MSCs can migrate to the site of injury (Marconi et al., 2012; Matthes et al., 2013), but are often transplanted into the vicinity of the lesioned nerve during the reconstructive surgery, resulting in a more concentrated effect and reduced local fibrosis (Wang et al., 2015; Cooney et al., 2016). MSCs are a natural source of pro-angiogenic and regenerative growth factors and act as a “living drug factory” for the healing nerve (Drzeniek et al., 2021). Their paracrine effects result in a better electrophysiological and functional outcome and micromorphological intactness of the healed nerve (Marconi et al., 2012; Matthes et al., 2013; Cooney et al., 2016; Bucan et al., 2019). Because the local microenvironment can affect MSC survival and function *in vivo*, protective carrier materials can shield MSCs from the harsh microenvironment, hold them in place and stimulate them to augment their growth factor secretion (Mao et al., 2017; Drzeniek et al., 2021). In recent years, it has been proposed to transplant MSC-derived vesicles, exosomes, instead of the entire living cell. These exosomes have also been shown to promote peripheral nerve regeneration, similarly to the parent cell (Bucan et al., 2019). Another recent study shows that exosomes from MSC-derived SC-like cells play a supporting role by protecting endogenous SCs from oxidative stress and promoting angiogenesis (Liu et al., 2022).

### 4.3 Functionalization Through Structural Material Design and Multimodal Strategies

Neurites can recognize surface geometry and their growth pattern follows local topography such as groves *in vitro* (Miller et al., 2001a). In order to recapitulate the microanatomical guiding sheaths, the intactness of which is outcome-determining according to the Sunderland classification, biomimetic NGC designs experiment with different anisotropic microstructures and micropatterns to provide more precise physical guidance at the cellular level *in vivo*. The field of architectured materials and NGCs is currently growing fast thanks to the rapid progress in spatiotemporally precise biofabrication methods, such as 3D bioprinting [reviewed by Dixon (Dixon et al., 2018)]. Many NGC designs discussed in this section combine several of the synthetic or natural polymers mentioned above and experiment with additional biological cues to achieve results comparable to the autograft.

#### 4.3.1 Bulk Filler Hydrogel

An easy way to functionalize the NGC lumen is with bulk hydrogel, which can provide a cell friendly environment, allows for easy incorporation of biochemical cues and soluble factors, but does not provide any topographical cues for directional determination and may even reduce the permissiveness of the NGC lumen for growing axons (Yoo et al., 2020; Drzeniek et al., 2021). Therefore, the opinion on bulk fillers is controversial and evidence which favors spatial patterning of hydrogels is growing (Yoo et al., 2020).

#### 4.3.2 Grooved Surfaces

The use of molded grooves on the inner NGC wall has been proposed with the rationale that grooves could not only provide anisotropic physical guidance to sprouting axons but also increase the surface area for cell attachment, cell-material interactions and potentially for controlled release of incorporated growth factors. In practice however, patterning 10 µm grooves on the inner NGC wall did not yield a relevant effect compared to an unpatterned chitosan NGC and failed to outperform the autograft control (Li et al., 2018). Similarly, in an older study a micropatterned inner lumen designed to regenerate a 10 mm rat sciatic nerve gap could only impact functional outcome when implemented in synergy with pre-seeded SCs (Rutkowski et al., 2004).

As an alternative to molded groves, longitudinally oriented collagen strips were 3D-printed on a porous PLCL membrane. It could be shown that the 3D-printed collagen lines led to a better axonal regeneration and remyelination than bulk collagen hydrogel filling, indicating that spatially controlled patterning of substrates that promote cell attachment can be a promising strategy that combines structural and substrate-mediated cues (Yoo et al., 2020). Similarly, in a very recent study a grooved PLCL conduit in combination with a patterned gradient of a laminin-derived peptide showed synergistic effects on aligned migration of SCs *in vitro* and significantly accelerated nerve recovery *in vivo* (Zhang et al., 2021).

It seems that the benefit of grooves manifests in combination with other factors such as biochemical cues or cells, or maybe acts on axons indirectly by influencing SC biology. Early *in vitro* studies had found that groove width versus depth differently influence the alignment of SCs versus neurites (Miller et al., 2001a; Miller et al., 2001b). *In vivo* studies often lack such comparisons and possibly successful use of grooves in NGC design would require more attention to detail and systematic optimization *in vivo*.

#### 4.3.3 Intraluminal Microchannels

**FIGURE 2:**
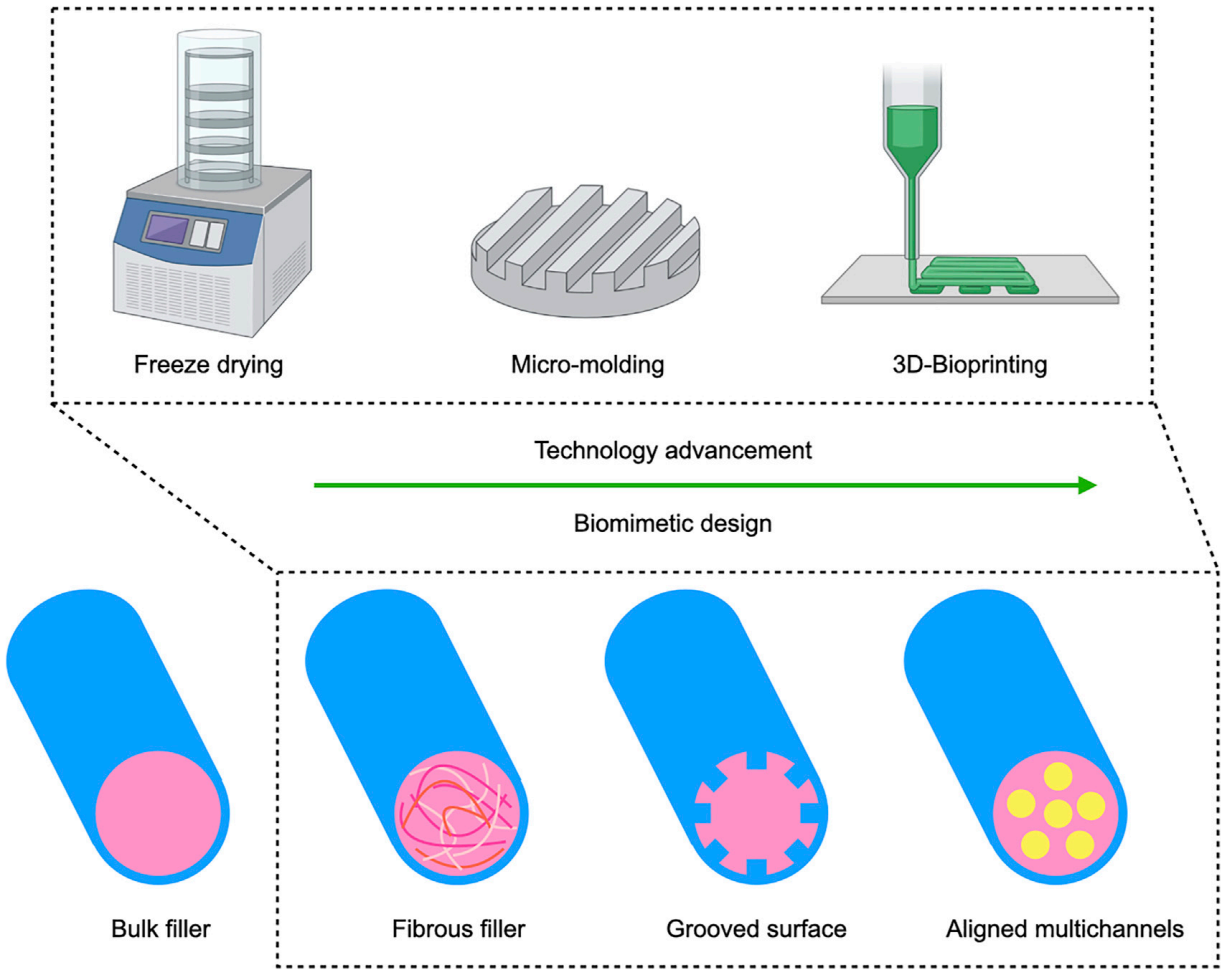
Advancement of structural cues for biomimetic NGC design: With the advancement of biofabrication techniques, structural cues for axon guidance are gaining relevance as an additional bioinstructive modality besides substrate mediated and soluble cues. The design of NGCs has evolved from very early nerve repair with hollow tubes to added anisotropic bulk gel or fibrous fillers. From the early 2000s on, research on longitudinally oriented fibers and grooves intensified and most recent studies increasingly investigate biomimetic multichannel conduits. 3D bioprinting allows to freely pattern multiple materials with or without cells and growth factors, thereby opening up new possibilities for multimodal implant fabrication.

Microchannels mimic the nerve fascicular (perineural) anatomy and prevent axon dispersion and can be introduced into the scaffold either by precise 3D bioprinting or through more conventional fabrication such as directional freeze drying or molding (Figure 2) (Hu et al., 2009; Wray et al., 2012; Zhang et al., 2022). Chang et al. (2017) used an NGC with micromolded intraluminal channels that resulted in increased axon diameter and myelin layer thickness compared to a control NGC without internal channels. While additional aligned nanofibers within the microchannels contributed only slightly to regeneration, adding an additional neurotrophic factor gradient, yielded an effect comparable to autograft. Similarly, the combination of a multichannel design with controlled release of 4-aminopyridine, a potassium channel blocker thought to improve nerve conduction in neurological disorders such as multiple sclerosis, was comparable to autograft in a 15 mm sciatic nerve gap rat model (Manoukian et al., 2021). Here the multichannels were fabricated by unidirectional freeze drying. For a simple yet clever spiral-shaped design, a grooved surface was rolled to fabricate multichannels which were combined with aligned nanofibers, but the structural cues alone failed to compete with the autograft control (Shah et al., 2019). The disadvantage of the aforementioned fabrication techniques is that the material architecture needs to be fabricated before therapeutic cells can be added to the conduit. In contrast to freeze drying, 3D bioprinting allows to integrate cells directly into the biomaterial. Recently, Zhang and colleagues used bioprinting to fabricate an advanced NGC (Zhang et al., 2022). SCs were incorporated into a methacrylated gelatin bioink and printed directly into the multi-channeled conduit.

These studies show that different fabrication techniques can be used to achieve an internal microchannel structure that mimics the natural anatomy of guiding sheaths. Emerging study design combining different modalities of bioactive cues stress the importance of synergistic biological and structural cues for NGC constructs that could promote nerve regeneration across larger gaps.

## 5 Challenges for Translating New NGC Designs Beyond Preclinical Studies

**FIGURE 3:**
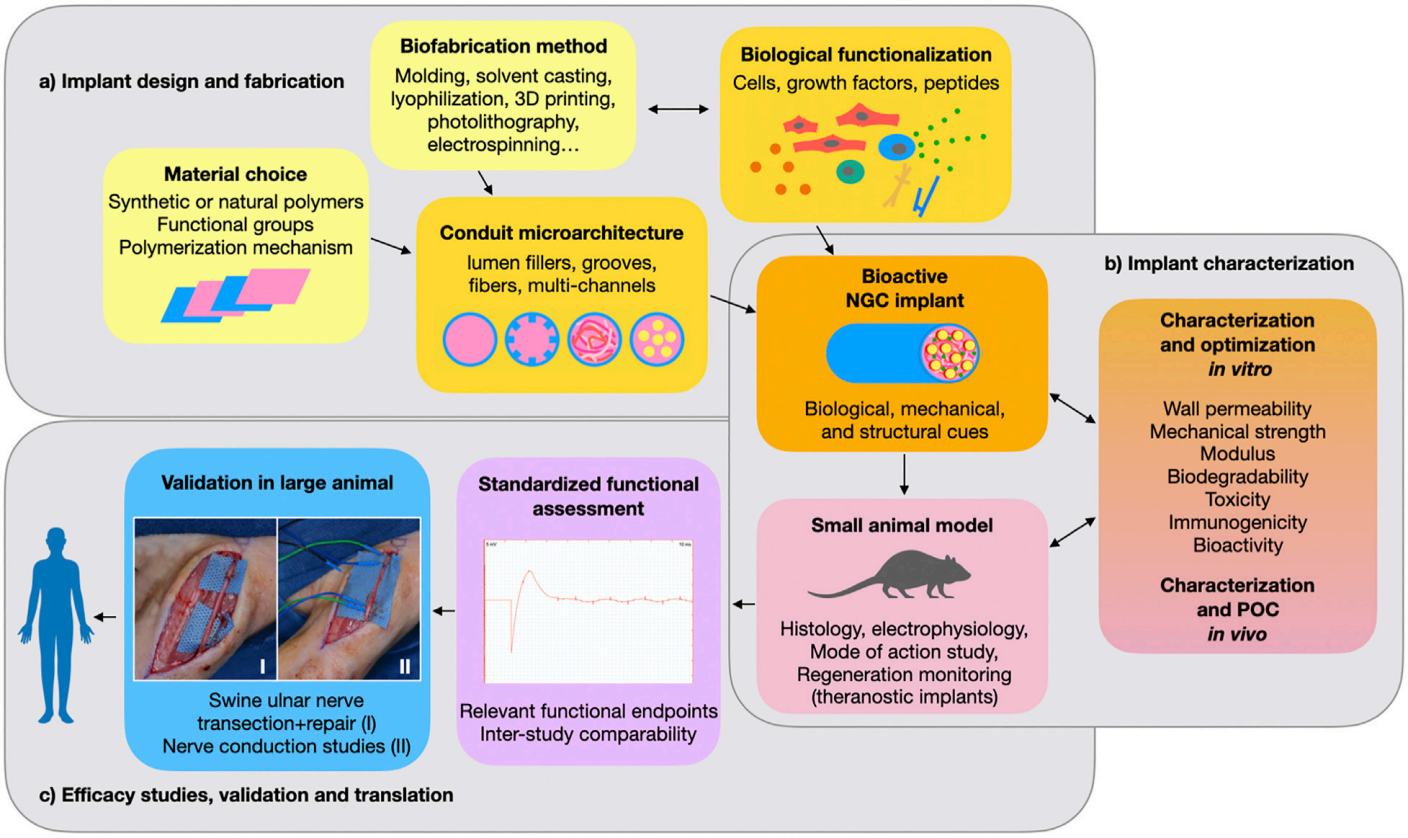
Preclinical development of a nerve conduit: **(A)** The choice of a suitable polymer and fabrication method determine the implant’s structural features and allow for biological functionalization with cells, proteins or peptides. **(B)** The resulting nerve guidance conduit (NGC) promotes nerve regeneration through a combination of biological, mechanical and structural cues. Several parameters are critical for safe and effective performance. These need to be thoroughly characterized both *in vitro* and *in vivo*. Most commonly, a rodent model is used for initial *in vivo* proof of concept (POC) and evaluation of safety and efficacy. **(C)** In order to identify the most promising implant designs and progress toward clinical application, performance of NGCs should be compared using standardized relevant endpoints and validated in large animal studies. Photographs I and II belong to the Gorantla lab.

Engineering a clinically usable alternative to the autograft for repair of large peripheral nerve gap injuries still poses an unsolved challenge. The first FDA-approved artificial NGCs were implants fabricated from biodegradable synthetic polymers that could bridge a short defect but failed at promoting axon regrowth across larger gaps. In an effort to increase the conduits’ bioactivity, the design of synthetic NGCs has progressed over the past decades from hollow tubes to the implementation of bioactive substrates and intraluminal structures for refined topographical guidance. In 2014, the FDA approved the first NGC with a porous lumen filler (NeuraGen^®^ 3D Nerve Guide Matrix), which is composed of a bioactive collagen-glycosaminoglycan blend and has substantially improved the conduit’s performance (Lee et al., 2012). Since then, preclinical studies have advanced to multimodal strategies that explore synergistic effects of substrate-, structure-, and soluble factor-mediated cues (Figure 3A). While some of these more intricate synthetic grafts have reached functional outcomes that are comparable to autografting in preclinical models, none have been approved for clinical use so far. As the conduits tested preclinically are becoming more complex and bioactive, their clinical acceptance declines compared to that of simpler and well understood hollow tube grafts. This could be in part because the more active components an implant has, the more challenging it becomes to translate it into a safe, well characterized, and reliable medical device.

A major translational challenge lies in choosing the correct preclinical *in vivo* model for translation to human application. Most preclinical studies show efficacy of their NGC in a rat sciatic nerve gap injury, usually of 10 mm gap size (Table 1). (Kaplan et al., 2015) showed that nearly 90% of the peripheral nerve gap repair studies are being conducted in rats and rabbits (78% rats and 12% rabbits). For translation, these studies have major limitations due to a species-specific neurobiological regenerative profile. Small animal studies have little relevance for translation without supporting large animal studies. Thus, more large animal studies are needed to identify truly promising conduits among the countless designs found in literature (Figure 3C).

**TABLE 1:** Chronological overview of NGC design and animal studies.

Year	Authors	NGC design	*In vivo* Model
1988	Dellon AL. et al. (Dellon and Mackinnon, 1988)	PGA	Monkey
1991	Archibald SJ. et al. (Archibald et al., 1991)	Collagen	Rat and monkey
1992	Guénard V. et al. (Guénard et al., 1992)	Semipermeable PAN/PVC conduit loaded with Schwann cells	Rat
1995	Tang JB. et al. (Tang et al., 1995)	Vein graft	Human
2000	Weber RA. et al. (Weber et al., 2000)	PGA	Human
2001	Miller C. et al. (Miller et al., 2001a)	Laminin-coated PDLA seeded with Schwann cells	Rat
2004	Rutkowski GE. et al. (Rutkowski et al., 2004)	PDLLA conduit seeded with Schwann cells	Rat
2005	Wang X. et al. (Wang et al., 2005)	Chitosan/PGA	Dog
2009	Hu X. et al. (Hu et al., 2009)	Collagen/Chitosan conduit with microchannels	Rat
2009	Rosson GD. et al. (Rosson et al., 2009)	PGA conduit	Human
2009	Whitlock EL. et al. (Whitlock et al., 2009)	Commercially available collagen versus allograft	Rat
2010	Durgam H. et al.	NGCs coated with PPy-PCL and PPy-PECA co-polymers	Rat
2011	Marcol W. et al. (Marcol et al., 2011)	Chitosan gel covered proximal nerve end	Rat
2011	Radtke C. et al. (Radtke et al., 2011)	Decellularized vein grafts filled with spider silk fibers	Sheep
2012	Lee JY. et al. (Lee et al., 2012)	Collagen conduit filled with collagen-glycosaminoglycan	Rat
2012	Wray LF. et al. (Wray et al., 2012)	Silk-based scaffold with hollow channels	—
2012	Giusti, G. et al. (Giusti et al., 2012)	Collagen versus allograft	Rat
2013	Berrocal YA. et al. (Berrocal et al., 2013)	Collagen conduits seeded with Schwann cells	Rat
2014	Matsumine H. et al. (Matsumine et al., 2014)	PLA-conduit and silicon conduit filled with Collagen	Rat
2014	Sahin C. et al. (Sahin et al., 2014)	Vein filled with minced nerve	Rat
2015	Kim B. et al.	PDMS (polydimethylsiloxane) microchannel scaffold with microwires (used as recording electrodes) embedded within the microchannels	Rat
2015	Musick KM. et al.	Microchannel electrode implants with silicone rubber and elastic thin-film metallization	Rat
2016	Eren F. et al. (Eren et al., 2016)	Vein graft with VEGF and stem cells	Rat
2016	MacEwan MR. et al.	GDNF loaded nerve guidance silicone-conduits with chronically implanted macro-sieve electrode	Rat
2017	Chang YC. et al. (Chang et al., 2017)	Multi-channeled scaffolds with electrospun nanofibers and NGF and BDNF	Rabbit
2017	Ko CH. et al. (Ko et al., 2017)	Bisvinyl sulfonemethyl (BVSM)-crosslinked gelatin conduit	Rat
2017	Yapici AK. et al. (Yapici et al., 2017)	Vascularized neurotube	Rat
2018	Li G. et al. (Li et al., 2018)	Chitosan conduit with micropatterned inner wall	Rat
2018	Neubrech F. et al. (Neubrech et al., 2018)	Chitosan wrap	Human
2018	Xia B. et al. (Xia and Lv, 2018)	PLLA-electrospun nanofibrous conduit loaded with VEGF and NGF	Rat
2019	Sun B. et al. (Sun et al., 2019)	Ppy-coated nerve guidance conduit	Rat
2019	Chen X. et al. (Chen et al., 2019)	Carboxylic graphene oxide-composited polypyrrole conduits loaded with mouse fibroblast cells and rat pheochromocytoma cells	Rat
2019	Shah MB. et al. (Shah et al., 2019)	Multichannel PCL spiral with aligned collagen nanofibers	Rat
2019	Singh A. et al. (Singh et al., 2019)	Polyurethane conduit filled with aligned chitosan-gelatin cryogel filler	—
2019	Vijayavenkataraman S. et al. (Vijayavenkataraman et al., 2019a)	PPy-b-PCL based conductive scaffolds seeded with peripheral neuronal cells	—
2019	Rbia N. et al. (Rbia et al., 2019)	Commercially available collagen versus allograft	Human
2020	Chang W. et al. (Chang et al., 2020)	Laminin cross-linked PCL/PEG spiral conduit with outer nanofibrous tube	Rat
2020	Donsante A. et al. (Donsante et al., 2020)	PCL conduit integrated with phase-change material loaded with NT-3 and ChABC	Rat
2020	Gisbert Roca F. et al. (Gisbert Roca et al., 2020)	Hyaluronic acid and silk fibroin conduits	Rat
2020	Itai S. et al. (Itai et al., 2020)	Chitosan-collagen hydrogel conduit loaded with Schwann cells	—
2020	Keshavarz M. et al. (Keshavarz et al., 2020)	Polycarbonate conduit with poly-ʟ-ornithine and double-walled carbon nanotubes	—
2020	Panzer KV. et al. (Panzer et al., 2020)	Tissue engineered bands of Büngner	—
2020	Yoo J. et al. (Yoo et al., 2020)	PLCL conduit with 3D printed collagen hydrogel	Rat
2020	Zhao Y. et al. (Zhao et al., 2020)	Polypyrrole/silk fibroin (PPy/SF) conductive composite scaffold seeded with Schwann Cells	Rat
2021	Carvalho CR. et al. (Carvalho et al., 2021)	Silk fibroin loaded with NGF and GDNF	Rat
2021	Huang Q. et al. (Huang et al., 2021)	Alginate-gelatin hydrogel with graphene mesh, loaded with netrin-1	Rat
2021	Kong Y. et al. (Kong et al., 2021)	HA-phenylboronic acid-poly (vinyl alcohol) -heparin hydrogel loaded with GDNF	Mouse
2021	Kornfeld T. et al. (Kornfeld et al., 2021b)	Spider silk-based artificial nerve graft	Sheep
2021	Manoukian OS. et al. (Manoukian et al., 2021)	Chitosan-halloysite nanotubes conduit loaded with 4-aminopyridin	Rat
2021	Zhang D. et al. (Zhang et al., 2021)	Grooved PLCL with laminin peptide gradient	Rat
2022	Zhang L. et al. (Zhang et al., 2022)	Schwann Cells 3D printed in gelatin-based microchannel conduit	—

Additionally, while many studies show efficacy of their design and use meaningful internal controls, they are hardly comparable with other studies in the field as there is little consensus on which readouts, time points and effect sizes are considered most relevant for clinical translation. The evaluation timepoint following nerve repair is crucial for thoroughly understanding the effect of the experimental technique on nerve regeneration. Brenner et al. (2008) demonstrated that while the nerve regenerative effect of tacrolimus is significant at 40 days, it is undetectable at 70 days. Similar results are found in a metanalysis by DeLeonibus et al., 2021.

As discussed in the “structural design” section of this review, synergistic biological effects of multimodal NGC functionalization are still poorly understood and would require extensive comparisons and scrutinous experimental design to optimize each component (Figures 3A,B). Furthermore, the already vast choice of individual bioactive factors such as cells and growth factors (cf. “biological functionalization” section) makes it difficult to choose the optimal combination. Therefore, rather than further expanding the choice of bioactive polymers, soluble factors, transplantable cells and structural designs, an important challenge for the NGC field lies in better understanding how different modes of action of each component can be synergized into combination therapies (Figure 3B). This can only be achieved in a collective effort, by improving and standardizing study design and readouts towards better inter-study comparability. Fortunately, recent studies in the field have recognized this issue and are combining more extensive *in vitro* comparisons and mode-of-action studies with a functional *in vivo* investigation.

Finally, in order to identify both biological mechanisms as well as translationally relevant readout, some attention should be brought to *in vivo* live monitoring of peripheral nerve regeneration. This could be achieved through theranostic implants that track the regenerative process *in vivo* (Figure 3). For example, some nerve interface studies use bioelectronics to track axon growth and electrophysiological performance (cf. Section 3.3.2), but other monitoring approaches are also conceivable and remain largely unexplored in the nerve regeneration field to date (Kim et al., 2015; Musick et al., 2015; Paggi et al., 2021).
